# Clinical efficacy and effect on neurotransmitter levels of Shaoma Zhijing granules in children with tic disorders

**DOI:** 10.3389/fped.2025.1549103

**Published:** 2025-04-16

**Authors:** Yanping Wang, Miao Jing, Ying Hua, Lin Zhang, Jianbiao Wang, Xiaochun Fan

**Affiliations:** ^1^ Department of Neurology, The Affiliated Wuxi Children’s Hospital of JiangNan University, Wuxi, Jiangsu Province, China; ^2^ Department of Emergency, Wuxi No.2 People’s Hospital, Wuxi, Jiangsu Province, China

**Keywords:** tic disorders, Shaoma Zhijing granules, tiapride, clinical efficacy, neurotransmitters

## Abstract

**Objective:**

To investigate the clinical efficacy of Shaoma Zhijing Granules in treating pediatric tic disorders and its impact on plasma neurotransmitter levels.

**Methods:**

Eighty pediatric patients with tic disorders were randomly divided into the Shaoma Zhijing group (40 cases) and the Tiapride group (40 cases). The Yale Global Tic Severity Scale (YGTSS) scores, clinical efficacy, and traditional Chinese medicine (TCM) syndrome evaluations were compared at baseline, 4 weeks, and 8 weeks after treatment. Ultra-performance liquid chromatography-tandem mass spectrometry (UPLC-MS/MS) was used to analyze the changes in plasma neurotransmitter levels before and after treatment.

**Results:**

Compared with baseline, both groups showed a decrease in YGTSS scores and TCM syndrome scores at 4 weeks and 8 weeks after treatment (*P* < 0.05). No statistically significant differences were observed between the two groups (*P* > 0.05). The effective rates were 90% for the Shaoma Zhijing group and 80% for the Tiapride group, with the incidence of adverse reactions being 2.5% and 15%, respectively. After 8 weeks, serum levels of glutamic acid (Glu), aspartic acid (Asp), dopamine (DA), norepinephrine (NE), and epinephrine (E) in both groups were significantly lower than baseline (*P* < 0.05), while gamma-aminobutyric acid (GABA) levels were significantly higher (*P* < 0.05). Significant differences were also observed between the two groups in DA, NE, E, and GABA levels after treatment (*P* < 0.05).

**Conclusion:**

Shaoma Zhijing Granules demonstrate significant clinical efficacy and good safety in treating pediatric tic disorders. They effectively improve symptoms, thereby contributing to the enrichment of the TCM diagnostic and treatment system for pediatric tic disorders.

## 1 Introduction

Tic disorders (TD) are common neurodevelopmental disorders in children (1). Clinically, they are characterized by involuntary motor and/or vocal tics. In recent years, the incidence of pediatric tic disorders has been steadily increasing, with a reported annual incidence rate of approximately 6.1% in China (2). These disorders can significantly affect children's learning, social interactions, and personality development (3).

The pathogenesis of TD is not fully understood. Most researchers believe that its onset may involve genetic factors, brain structural abnormalities, and neurotransmitter imbalances due to brain dysfunction (4, 5). Neurotransmitters, which primarily facilitate information transmission within the nervous system, have been extensively studied in children with TD. Current research focuses on biogenic amines [dopamine [DA], epinephrine [E], norepinephrine [NE]] and amino acids (glutamic acid [Glu], aspartic acid [Asp], gamma-aminobutyric acid [GABA], histidine [His], tryptophan [Trp]). Excessive DA activity or DA receptor hypersensitivity is a widely accepted mechanism underlying TD. Based on this theory, the 2020 Chinese Expert Consensus on the Diagnosis and Treatment of Pediatric Tic Disorders recommends tiapride, a classic DA receptor blocker, as a first-line treatment (6). Several studies have confirmed the efficacy of tiapride in treating pediatric TD (7–9). However, long-term use may cause side effects such as like dizziness, drowsiness, fatigue, and weight gain, and symptoms often relapse after discontinuation, which can reduce patient compliance. From a traditional medicine perspective, TD is often categorized in traditional Chinese medicine (TCM) as “liver wind,” “convulsions,” or “chronic convulsions.” Shaoma Zhijing Granules (SMZJG, also known as 5-ling granule, or TSupport/T92 under U.S. development), is an innovative TCM formulation, combines multiple herbs to calm liver yang, extinguish wind and stop spasms, clear fire and resolve phlegm, and enhancing efficacy while reducing toxicity. This study included 80 children with TD in a randomized controlled trial to compare the efficacy of Shaoma Zhijing granules and tiapride, as well as their effects on plasma neurotransmitter levels. Tiapride was included as a positive control due to its proven efficacy in treating TD (9, 10).

## 2 Materials and methods

### 2.1 Clinical data collection

**Figure 1:**
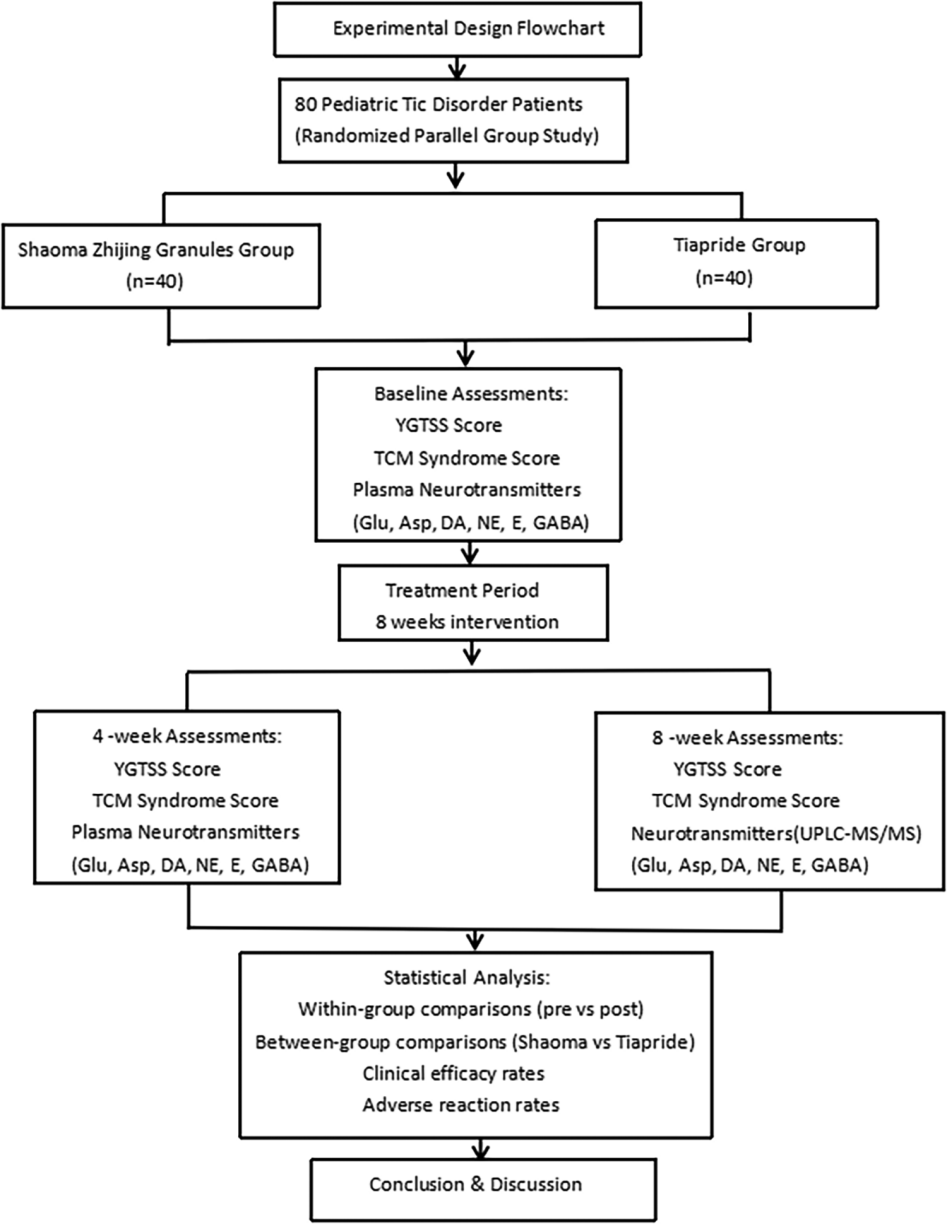
Experimental design flowchart.

Children with TD who visited the outpatient department of Wuxi Children's Hospital between November 2022 and October 2023 were selected for the study. A total of 80 participants were enrolled and randomly assigned in a 1:1 ratio to the experimental group (Shaoma Zhijing granule, 40 cases) or the control group (Tiapride, 40 cases) using a random number table. The detailed allocation process is presented in the flowchart (Figure 1). All participants received monotherapy without any concomitant medications. Importantly, outcome assessors were blinded to treatment allocation throughout the study. In the Shaoma Zhijing group, there were 26 boys and 14 girls, aged 5–14 years. Of these, 12 were 5–7 years old, 19 were 8–10 years old, and 9 were 11–14 years old, with an average age of 8.30 ± 1.70 years. The disease duration ranged from 6 months to 4 years, with an average of 17.70 ± 7.65 months. In the Tiapride group, there were 27 boys and 13 girls, aged 6–13 years. Of these, 10 were 5–7 years old, 19 were 8–10 years old, and 11 were 11–14 years old, with an average age of 8.85 ± 1.92 years. The duration of illness ranged from 6 months to 5 years, with an average of 17.23 ± 8.70 months. There were no statistically significant differences in gender, age, duration of illness comorbidities, difficulty falling asleep, or parental mental disorders between the two groups (*P* > 0.05), as shown in Table 1. All selected children underwent rigorous clinical symptom screening and were diagnosed by two neurologists and two TCM doctors. The diagnostic criteria referred to the “Expert Consensus on the Diagnosis and Treatment of Tic Disorders in Children (2020 Edition)” (6), the 5th edition of the “Diagnostic and Statistical Manual of Mental Disorders” (DSM-5) (11), and the diagnostic standards from the “Clinical Practice Guidelines for Traditional Chinese Medicine Pediatrics: Tic Disorders” (12). This study was approved by the Ethics Committee of Wuxi Children's Hospital (Ethics No.: WXCH2022–09-079). All participants and their families signed informed consent forms.

**Table 1:** Baseline characteristics of participants between the two groups.

Characteristic	Shaoma Zhijing group	Tiapride group	Statistical test	*χ*^2^/*t* value	*P*
Gender, *n* (%)			Chi-square test	0.018	0.891
Male	26 (65%)	27 (67.5%)			
Female	14 (35%)	13 (32.5%)			
Age(years, mean ± SD)	8.30 ± 1.70	8.85 ± 1.92	Independent *t*-test	*t* = −1.36	0.178
Duration of illness (months, mean ± SD)	17.70 ± 7.65	17.23 ± 8.70	Independent *t*-test	*t* = 0.26	0.796
Comorbidities, *n* (%)			Chi-square test	0.088	0.767
ADHD	8 (20%)	7 (17.5%)			
Siblings, *n* (%)			Chi-square test	0.156	0.693
Yes	5 (12.5%)	6 (15%)			
No	35 (87.5%)	34 (85%)			
Difficulty falling asleep, *n* (%)			Chi-square test	0.087	0.768
Yes	10 (25%)	9 (22.5%)			
No	30 (75%)	31 (77.5%)			
Parental mental disorders, *n* (%)			Fisher's exact test		1.0
Yes	3 (7.5%)	4 (10%)			
No	37 (92.5%)	36 (90%)			

*n*, number of participants; SD, standard deviation; *P*, *P*-value (statistical significance); ADHD, attention-deficit/hyperactivity disorder.

### 2.2 Inclusion and exclusion criteria

Inclusion Criteria: (1) Meets the DSM-5 diagnostic criteria for tic disorders, with frequent tic symptoms lasting for more than 6 months; (2) Meets the TCM diagnostic criteria for hyperactive liver wind syndrome. The diagnostic criteria are as follows: Primary symptoms: frowning, blinking, grimacing, mouth opening, shoulder shrugging, head shaking, arm swinging, leg kicking, loud shouting, frequent and intense tics, or vocal tics with obscene language. Secondary symptoms: irritability, hot palms and soles, restless sleep, dry stools, dark yellow urine, red tongue with a thin yellow or greasy yellow coating. To diagnose Hyperactive Liver Wind Syndrome with Phlegm-Fire internal disturbance, at least two primary symptoms and two secondary symptoms must be present, along with tongue and pulse diagnosis.

Explanation of TCM Terms:

Hyperactive Liver Wind Syndrome: In TCM, this syndrome refers to an overactivity of liver yang leading to internal wind, manifesting as involuntary movements and tics, often associated with irritability and restlessness.

Liver Wind: A TCM concept referring to internal wind movement caused by liver dysfunction, primarily manifested as tremors, tics, or other involuntary movements.

Phlegm-Fire: A condition characterized by the accumulation of phlegm and excessive heat in the body, resulting in symptoms like irritability, restless sleep, and a greasy yellow tongue coating.

(3) Aged between 5 and 18 years, regardless of gender; (4) Provides informed consent and can accept the prescribed treatment plan and follow-up requirements.

Exclusion Criteria: (1) Does not meet the inclusion criteria; (2) Clearly diagnosed with epilepsy, chorea, autism spectrum disorder (ASD), intellectual disability (ID), athetosis, Wilson's disease, or autoimmune diseases; (3) Children with severe diseases involving the heart, brain, liver, kidneys, or hematopoietic system; (4) Clinically significant abnormal electrocardiogram; (5) Known allergy to TCM or its components; (6) Other serious diseases.

### 2.3 Treatment methods

Shaoma Zhijing granules (Tasly Pharmaceutical Group Co., Ltd., National Medicine Standard Z20190022, specification: 2.5 g/bag) were taken orally. The main ingredients are Radix Paeoniae Alba (Bai Shao), Rhizoma Gastrodiae (Tian Ma), Fructus Tribuli (Ji Li), Ramulus Uncariae (Gou Teng), Ganoderma (Ling Zhi), Caulis Polygoni Multiflori (Shou Wu Teng), Semen Ziziphi Spinosae (Suan Zao Ren), vinegar-prepared Fructus Schisandrae (Wu Wei Zi), Fructus Gardeniae (Zhi Zi), Rhizoma Arisaematis cum bile (Dan Nan Xing), and Radix Scutellariae (Huang Qin). For children aged 5–12 years: 5 g per dose (2 bags), three times a day; For children aged 13–18 years: 7.5 g per dose (3 bags), three times a day. Tiapride (Jiangsu Enhua Pharmaceutical Co., Ltd., specification: 100 mg/tablet) was administered at a dose of 50–100 mg per day, divided into 2–3 doses. After 1–2 weeks, the dose was gradually increased based on the patient's condition and age to 200–400 mg per day, with regular follow-up. The treatment and observation period for both groups was 8 weeks.

### 2.4 Observation indicators

#### 2.4.1 Tic severity

The severity of tic symptoms in both groups of children was analyzed using the Yale Global Tic Severity Scale (YGTSS) before treatment, 4 weeks, and 8 weeks after treatment (13). The YGTSS assessment was conducted by two experienced neurologists who received standardized training prior to the study. The evaluators were blinded to the participants’ treatment assignments throughout the assessments to ensure objectivity and consistency. Main Indicators: Tic scores are composed of five elements: type, number, frequency, intensity, interference, and complexity of motor and vocal tics. Each element is scored from 0 to 5. The total score for motor tics and vocal tics is 25 points each. Secondary Indicators: The YGTSS also assesses the level of social impairment caused by tics. This refers to the distress caused by tics in areas such as self-esteem, family life, social interactions, and academic or work performance. The impairment is evaluated on a 6-point scale (none to severe), corresponding to scores from 0 to 50, with higher scores indicating greater social impairment.

TCM Syndrome Grading and Quantification Standards:

Primary Symptoms: The primary symptoms consist of five items: frowning and blinking, opening the mouth and grimacing, shaking the head and shrugging shoulders, flinging hands and kicking legs, and making strange noises or uttering foul language.

(1) No symptoms: 0 points;
(2) Mild symptoms, such as only frowning or blinking, or only opening mouth or grimacing: 1 point each;
(3) Frequent occurrence: 2 points each;
(4) Constant occurrence: 3 points each.

Secondary Symptoms: The secondary symptoms include five items: irritability, hot palms and soles, restless sleep, dry stools, and dark yellow urine.

(1) No symptoms: 0 points
(2) Presence of symptom: 1 point each.

#### 2.4.2 Efficacy evaluation

The clinical efficacy of the two groups of children was evaluated using the YGTSS before treatment, and 8 weeks after treatment. The clinical efficacy is categorized into four levels: (1) Cured: Symptoms and signs have basically disappeared within 8 weeks of medication, with a total score reduction of ≥90%, and no recurrence for more than 1 month; (2) Significantly Effective: Tics have significantly reduced within 8 weeks of medication, with a total score reduction of ≥70% but <90%. (3) Effective: Tics have somewhat improved within 8 weeks of medication, with a total score reduction of ≥30% but <70%; (4)Ineffective: No improvement or worsening of tics within 8 weeks of medication, with a total score reduction of <30%;

The total effective rate is calculated as follows:

Total Effective Rate = (Number of cured cases + Number of significantly effective cases + Number of effective cases Total number of cases) × 100%

#### 2.4.3 Plasma neurotransmitter level detection

All enrolled children were instructed to maintain consistent daily habits before sample collection, including following a light diet, avoiding major lifestyle changes, minimizing the intake of caffeinated stimulants (such as coffee, cola, etc.), and refraining from medications that could influence neurotransmitter levels. They were also advised to ensure adequate sleep before sample collection. Venous blood samples (3 ml) were collected in the early morning after fasting from both groups at initial enrollment and after 8 weeks of treatment. The samples were placed in heparin anticoagulant tubes and centrifuged at 10,000 rpm for 10 minutes. After centrifugation, the plasma was stored in a freezer at −20°C. The levels of neurotransmitters [DA, GLU, GABA, Asp, NE, E, Trp, His, Tyrosine (Tyr)] in the plasma were analyzed using ultra-high-performance liquid chromatography-tandem mass spectrometry (UHPLC-MS/MS). Quantitative analysis was conducted using isotope-labeled internal standards to ensure measurement accuracy and reliability. For each neurotransmitter, calibration curves were generated using certified reference standards at concentrations spanning the physiological range. Quality control measures included: (1) analysis of low-, medium-, and high-concentration QC samples for internal quality control (IQC), and (2) participation in External Quality Assessment (EQA) programs to assess both intra- and inter-laboratory variability.

#### 2.4.4 Statistical analysis

Data analysis was performed using SPSS 26.0 statistical software. A *t*-test was used for the measurement data of the two groups, with the measurement data expressed as (*x* ± s). The chi-square (χ^2^) test was used for categorical data. A *p*-value of less than 0.05 was considered statistically significant.

## 3 Results

### 3.1 Comparison of YGTSS scores before and after treatment in the two groups

**Figure 2:**
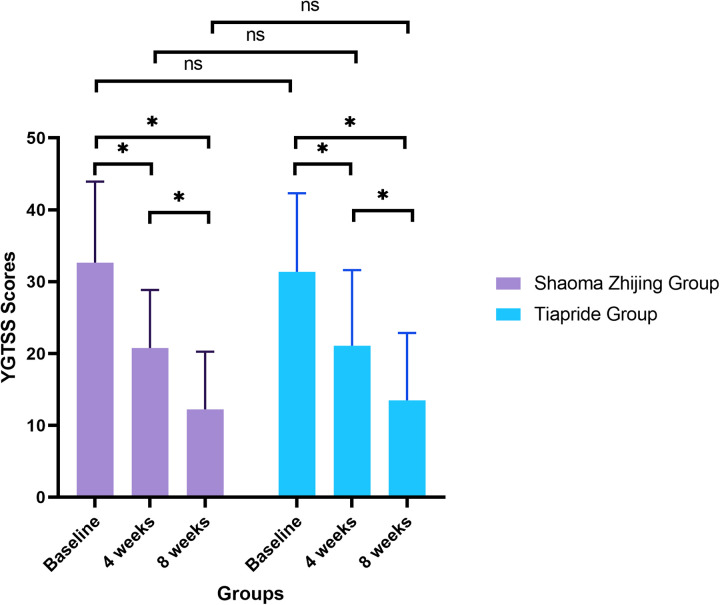
Comparison of YGTSS scores in two groups at different times (*x* ± s).

YGTSS scores decreased significantly in both groups during the treatment period (Shaoma group: F = 49.1, *P* < 0.05; Tiapride group: F = 30.32, *P* < 0.05). At baseline, no significant difference was found between the two groups (Shaoma: 32.65 ± 11.28 vs. Tiapride: 31.35 ± 10.96; *t* = 0.523, *P* = 0.603). After 4 weeks of treatment, scores in both groups were significantly lower than baseline (*P* < 0.05), with no significant intergroup difference (*t* = −0.155, *P* = 0.877). By week 8, scores had further declined (Shaoma: 12.23 ± 8.04; Tiapride: 13.48 ± 9.38), showing significant improvement compared to week 4 (*P* < 0.05), while intergroup differences remained statistically non-significant (*t* = −0.640, *P* = 0.524). (See Figure 2).

### 3.2 Comparison of TCM syndrome efficacy before and after treatment in the two groups

**Figure 3:**
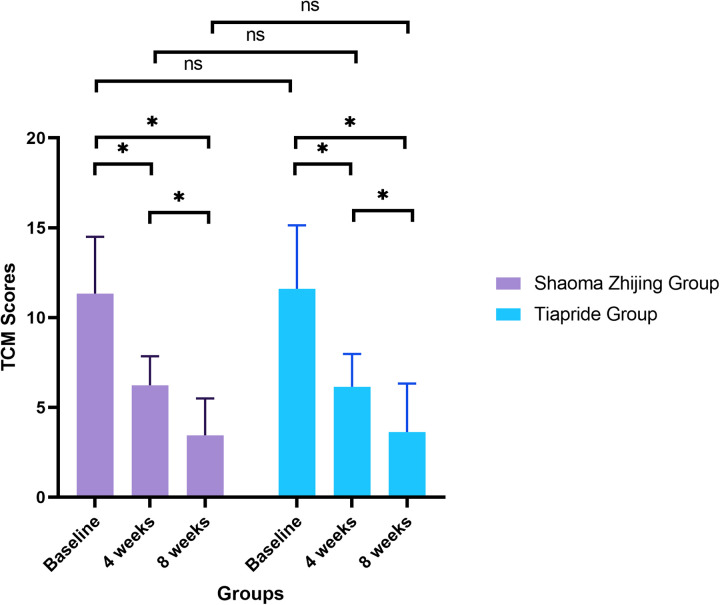
Comparison of TCM syndrome scores in two groups at different times (*x* ± s).

TCM syndrome scores demonstrated significant improvement in both groups over time (Shaoma group: F = 106.22, *P* < 0.05; Tiapride group: F = 89.33, *P* < 0.05). Baseline scores were comparable between groups (Shaoma: 11.33 ± 3.17 vs. Tiapride: 11.60 ± 3.54; *t* = −0.366, *P* = 0.716). By week 4, scores in both groups had decreased markedly from baseline (*P* < 0.05), with no significant difference between groups (*t* = 0.194, *P* = 0.847). At week 8, scores reached their lowest values (Shaoma: 3.45 ± 2.05; Tiapride: 3.63 ± 2.70), demonstrating significant improvement compared to week 4 (*P* < 0.05), while intergroup differences remained non-significant (*t* = −0.327, *P* = 0.745) (see Figure 3).

### 3.3 Comparison of clinical efficacy between the two groups

The efficacy rates in the Shaoma Zhijing Granule group for the 5–7, 8–10, and 11–14 age groups were 91.7%, 89.5%, and 88.9%, respectively, with an overall efficacy rate of 90%. In the Tiapride group, the efficacy rates for the same age groups were 80%, 78.9%, and 81.8%, with an overall efficacy rate of 80%. A statistically significant difference was observed in the overall efficacy rates between the two groups (*P* < 0.05) (see Table 2).

**Table 2:** Comparison on clinical efficacy between two groups.

Groups	Age (y, *n*)	Cured	Significantly Effective	Effective	Ineffective	Effective rate(%)	Overall effective rate (%)
Shaoma Zhijing group	5–7 (*n* = 12)	2	3	6	1	91.70%	90%
	8–10 (*n* = 19)	3	5	9	2	89.50%	
	11–14 (*n* = 9)	2	2	4	1	88.90%	
Tiapride group	5–7 (*n* = 10)	1	3	4	2	80%	80%
	8–10 (*n* = 19)	3	4	8	4	78.90%	
	11–14 (*n* = 11)	1	3	5	2	81.80%	
*X^2^*							3.92
*P*							0.048

*n*, number of participants; y, years; %, percentage; *X^2^*, Chi-square value.

### 3.4 Serum neurotransmitter levels before and after treatment in the two groups

**Figure 4:**
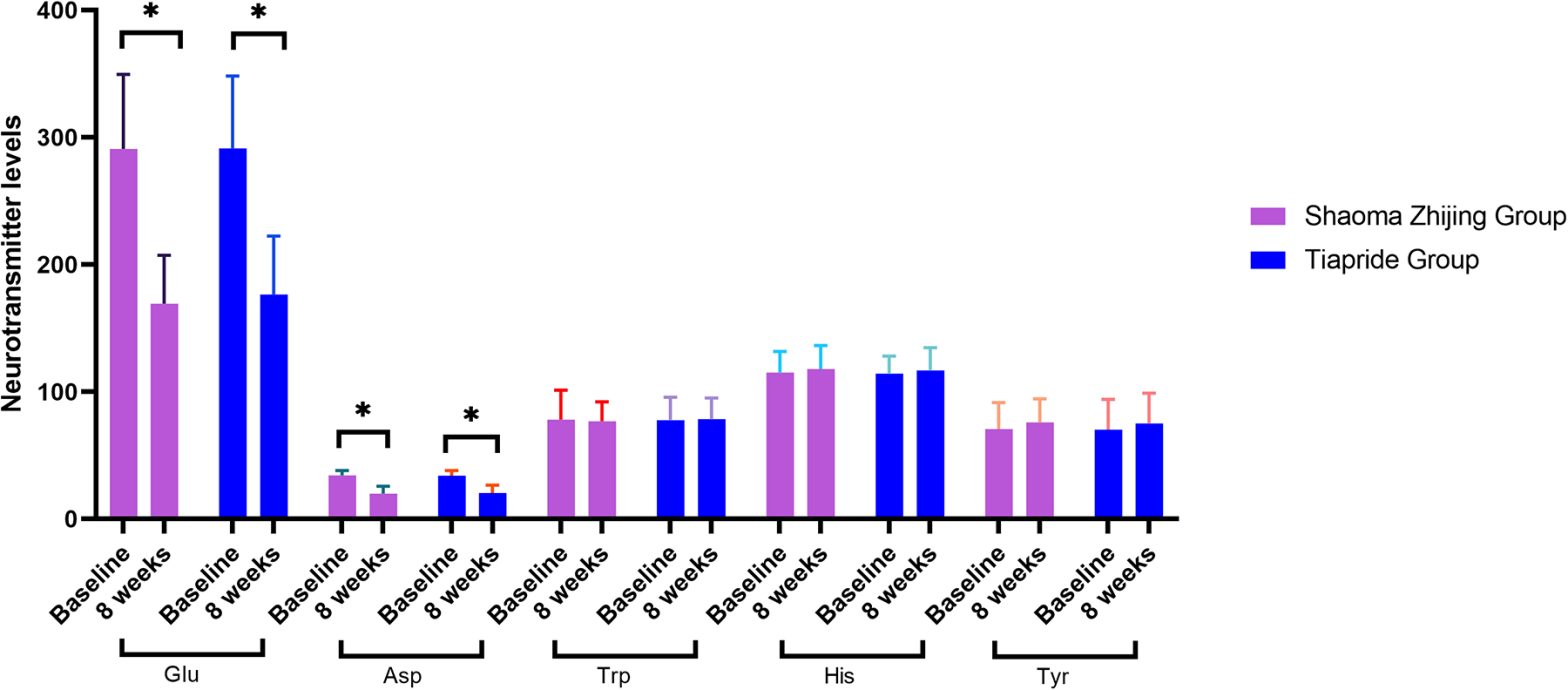
Comparison of serum neurotransmitter levels in two groups.

**Figure 5:**
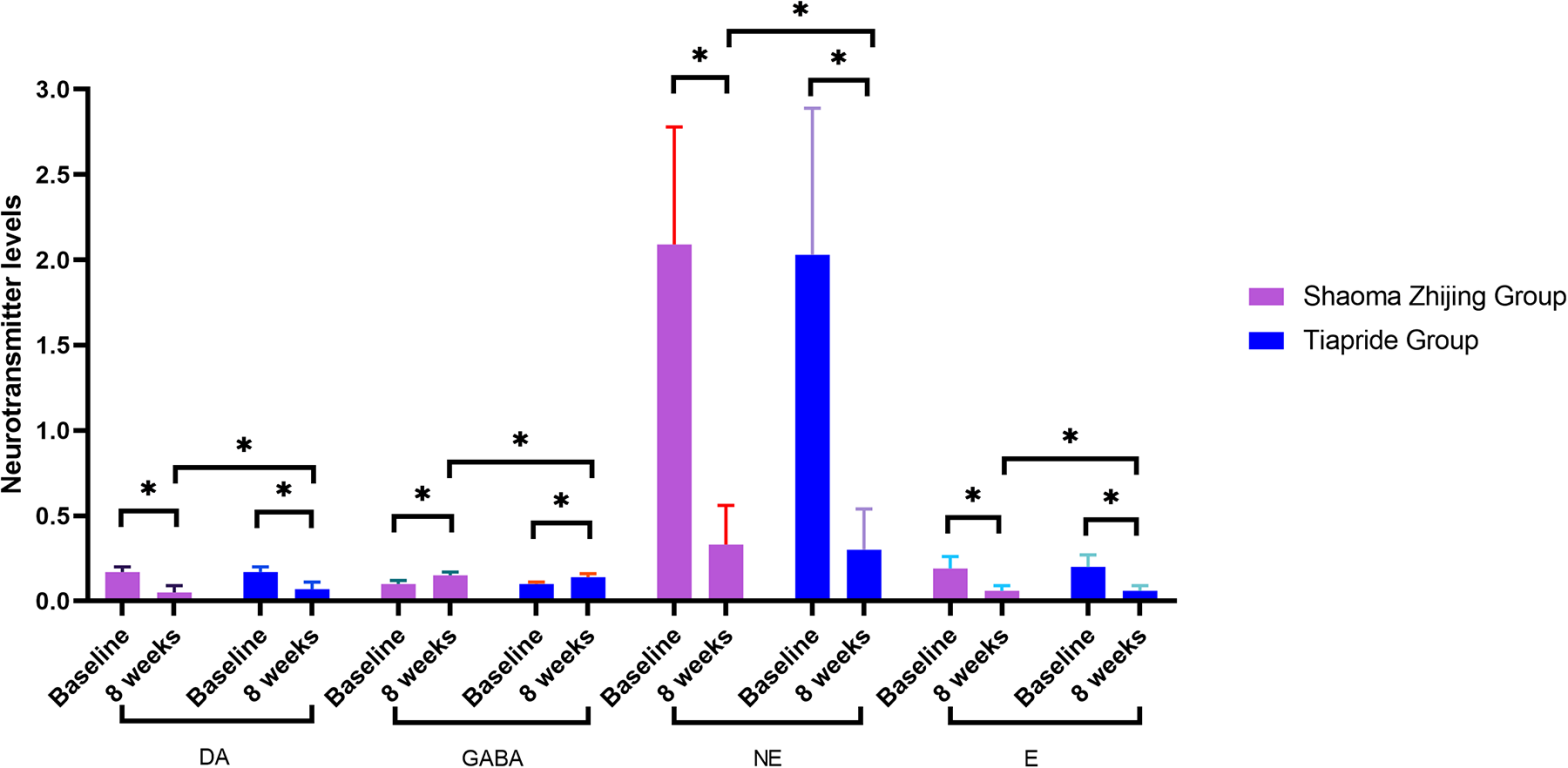
Comparison of serum neurotransmitter levels in two groups.

As demonstrated in Figures 4, 5, baseline neurotransmitter levels (Glu, Asp, DA, NE, E, GABA) were comparable between groups (all *P* > 0.05). Post-treatment analysis revealed significant reductions in excitatory neurotransmitters (Glu: Shaoma group, 290.8 → 169.4 μmol/L; Tiapride group, 291.2 → 176.5 μmol/L; Asp: Shaoma group, 34.4 → 19.9 μmol/L; Tiapride group, 34.1 → 20.5 μmol/L) and catecholamines (DA, NE, and E; all *P* < 0.05 vs. baseline), while GABA levels increased significantly (Shaoma group: 0.10 → 0.15 μmol/L; Tiapride group: 0.10 → 0.14 μmol/L; both *P* < 0.05). Notably, the Shaoma group showed greater reductions in DA (*t* = −2.233, *P* = 0.028) and a more pronounced increase in GABA (*t* = 2.155, *P* = 0.034) compared to the Tiapride group. In contrast, aromatic amino acids (Trp, His, Tyr) remained stable throughout the treatment (all intergroup *P* > 0.05), as visually confirmed in Figure 4.

### 3.5 Comparison of adverse reactions between the two groups of patients

In the Tiapride group, there were 6 cases of adverse reactions, including 2 cases of fatigue, 2 cases of dizziness, 1 case of drowsiness, and 1 case of dry mouth. In the Shaoma Zhijing group, there was 1 case of adverse reaction, which presented as nausea. The incidence of adverse reactions in the Shaoma Zhijing group and the Tiapride group was approximately 2.5% and 15%, respectively. There was a statistically significant difference between the two groups (*P* < 0.05) (see Table 3).

**Table 3:** Adverse reaction comparison (P/%).

Groups	Fatigue	Dizziness	Drowsiness	Nausea	Dry mouth	Overall incidence (%)
Shaoma Zhijing group (*n* = 40)	0	0	0	1	0	1 (2.5%)
Tiapride group (*n* = 40)	2	2	1	0	1	6 (15%)
*X^2^*						3.914
*P*						0.048

*n*, Number of participants; *X*^2^, Chi-square value; *P*, *P*-value (statistical significance); %, Percentage.

## 4 Discussion

Tic disorders are common neurodevelopmental disorders that typically begin in childhood, characterized by diverse symptoms and recurrent episodes. These disorders can impact a child's learning, social interactions, and personality development. Therefore, early and appropriate pharmacological treatment is essential for the effective comprehensive management of tic disorders in children.

As is well known, although tiapride has demonstrated good clinical efficacy, it is associated with numerous adverse reactions and a high risk of relapse after discontinuation, reducing tolerability and compliance in children, thereby limiting its clinical application. TCM, with its millennia-long history in treating pediatric disorders, has gained recognition for its multifaceted treatment modalities with minimal adverse effects (14). TCM classifies tic disorders under the category of “Liver Wind,” attributing them to hyperactive yang, yin deficiency, and external wind stirring (15, 16.) TCM views the complex, variable symptoms and recurrent nature of this disorder as consistent with the pathogenic characteristics of wind and phlegm.

This study found that Shaoma Zhijing granules improved YGTSS scores after 4 and 8 weeks of treatment compared to baseline. Moreover, the TCM syndrome scores in the Shaoma Zhijing group were significantly lower than those in the control group after treatment (*P* < 0.05). The clinical efficacy rate was slightly higher than that of the Tiapride group, which can likely be attributed to Shaoma Zhijing granules being an innovative Chinese herbal medicine for treating pediatric tic disorders. Selected patients were diagnosed with hyperactive liver wind and internal disturbance of phlegm-fire, a TCM pattern that may account for the observed superiority over the Tiapride group. There was no statistically significant difference in efficacy across age groups within each treatment group, indicating that age did not affect clinical outcomes. The medicinal ingredients of Shaoma Zhijing Granules exhibit unique synergistic effects. Pharmacological evidence suggests that the active components of these herbs interact with specific receptors and signaling pathways implicated in tic disorders. For instance, both Uncaria (Gou Teng) and Fructus Tribuli (Ji Li) have been traditionally used to calm the liver, relieve depression, extinguish wind, and stop convulsions. Modern research indicates that Uncaria may exert these effects primarily through the modulation of GABAergic and dopaminergic systems, with its active components (e.g., rhynchophylline) demonstrating significant neuroprotective and anticonvulsant properties. Similarly, Fructus Tribuli likely contributes to these therapeutic actions through its anti-inflammatory and antioxidant activities. Rhizoma Arisaematis cum bile (Dan Nan Xing) possesses anticonvulsant properties, potentially mediated by its effects on glutamate receptors, which Caulis Polygoni Multiflori (Shou Wu Teng) dispels wind and promotes circulation, likely through anti-inflammatory and neuroprotective mechanisms. Modern pharmacological studies indicate that Radix Paeoniae Alba (Bai Shao) has anti-inflammatory, analgesic, anti-myocardial ischemia, antidepressant, and immunomodulatory effects (17). Gastrodia (Tian Ma) and Uncaria (Gou Teng) are used as a pair of medicines, with their main components being gastrodin and rhynchophylline, which have excellent antioxidant and anti-cell damage effects (18). Numerous studies have highlighted the therapeutic potential of gastrodin in alleviating TS, primarily through its regulation of the DA system (19, 20). Based on this, Semen Ziziphi Spinosae (Suan Zao Ren) was specifically added to the formula. Its main active component, jujuboside A, exhibits a wide range of pharmacological effects, particularly in sedation, sleep promotion, and neuroprotection (21). At low-dose jujuboside A enhances its sedative and hypnotic effects by upregulating the expression of GABA receptors (α1, α5, β2), thereby alleviating sleep disorders associated with tic disorders (22). The introduction of jujuboside A is intended to further reduce nervous tension and improve tic symptoms. Overall, the combination of medicinal ingredients in Shaoma Zhijing Granules is well-balanced, effectively relieving nervous tension, improving tic-related TCM syndromes, and demonstrating strong clinical efficacy.

Most researchers believe that the frontal cortex, thalamus, basal ganglia, and hippocampus are the primary pathological regions of this disease (23–25). Neurotransmitter abnormalities within the cortico-basal ganglia-thalamo-cortical circuit play a critical role in the pathogenesis of tic disorders. Various neurotransmitters, including DA, NE, GABA, and Glu systems, are active in these circuits (26). The imbalance between inhibitory and excitatory signals in this circuit is considered the molecular mechanism underlying the generation of tics and related symptoms. In recent years, genetic, pharmacological, and brain imaging studies have indicated that histaminergic pathways may be associated with TD (27, 28). Additionally, other research suggests that His may be involved in the pathogenesis of tic disorders by regulating the release of neurotransmitters such as DA, GABA, 5-Hydroxytryptamine (5-HT), and Glu at both presynaptic and postsynaptic levels (29). Zhang et al. (30) found that children with TD have reduced inhibitory neurotransmitter GABA in both direct and indirect projection pathways in the brain. This results in insufficient inhibition of excitatory neurons in the thalamocortical region, leading to increased levels of excitatory amino acids such as Glu and ASP, and decreased levels of GABA, thereby contributing to the manifestation of tic symptoms. Currently, there is a growing number of studies on neurotransmitters in children with tic disorders, with primary specimens being blood, urine, and cerebrospinal fluid. While cerebrospinal fluid, as a brain buffer, more accurately reflects neurotransmitter levels, its collection is often limited by strict requirements and low patient compliance, making it less preferred in this study. Urine, being a metabolic waste product, primarily reflects neurotransmitter metabolites and was therefore excluded from this study. In contrast, blood samples are easily obtained in clinical settings, have higher patient compliance, and their plasma neurotransmitter levels can provide a reliable reflection of the body's condition (31). Furthermore, several studies have shown that plasma neurotransmitter levels in children with tic disorders are abnormal (32, 33). The results of this study show that after 8 weeks of treatment, serum levels of GLU, Asp, DA, NE, and E in both groups were lower compared to before treatment, while GABA levels were higher. Notably, the Shaoma Zhijing granules group demonstrated significantly greater reductions in DA, NE, and E, along with higher GABA elevation, than the tiapride group. These findings suggest that Shaoma Zhijing Granules may effectively treat tic disorders by modulating GABAergic and dopaminergic pathways to rebalance excitatory-inhibitory activity in the cortico-basal ganglia-thalamo-cortical circuit. The slightly superior clinical efficacy compared to tiapride may result from its broader neuromodulatory effects on DA, GABA, and NE systems, while neither treatment affected His, Trp, or Tyr levels. The observed neurotransmitter changes, which potentially correlated with symptom improvement, indicate potential therapeutic value that warrants further clinical investigation.

Our research on drug safety also found that the incidence of adverse reactions with Shaoma Zhijing Granules is significantly lower than that with tiapride. It is well known that long-term use of tiapride can cause side effects such as dizziness, fatigue, drowsiness, and weakness. For children with mild to moderate tic disorders, as well as for families concerned about the long-term side effects of tiapride, Shaoma Zhijing Granules may serve as a safer and more tolerable treatment option. Although this study did not specifically examine the combined use of Shaoma Zhijing Granules and tiapride, some clinicians have observed in practice that combining the two may yield in more significant therapeutic effects for children with moderate to severe tic disorders. However, the potential benefits of this combination therapy require further clinical validation through well-designed studies. The herbal components of Shaoma Zhijing Granules are generally safe and reliable, with few adverse reactions, rapid onset of action, and stable therapeutic effects, thereby providing a novel pharmacological approach for the treatment of tic disorders. It is important to noted that TD are closely related to a child's psychological state. Therefore, integrating necessary psychological support and cognitive interventions during pharmacological treatment may enhance overall efficacy. This study has several limitations, including a relatively small sample size, short observation period, and insufficient consideration of psychological and environmental factors that may influence tic disorders. The 8-week treatment period, while adequate for observing initial therapeutic effects, is relatively brief for assessing long-term outcomes, such as potential symptom recurrence after discontinuation of Shaoma Zhijing Granules. Future studies should include extended follow-up periods to investigate whether symptom rebound occurs post-treatment and to evaluate the sustainability of therapeutic effects. Additionally, larger sample sizes, improved control over external influencing factors, and the integration of clinical studies with animal experiments are essential to further validate the efficacy and safety of Shaoma Zhijing Granules. These measures will enhance the reliability of the findings and provide stronger evidence to support its clinical application in the treatment of tic disorders.

In conclusion, Shaoma Zhijing Granules can improve TCM syndromes, alleviate tic symptoms in children with tic disorders, enhance clinical efficacy, regulate neurotransmitter levels, and result in fewer adverse reactions. These findings contribute to the advancement of TCM-based treatments for pediatric tic disorders and highlight the potential for broader clinical application.

## Data Availability

The original contributions presented in the study are included in the article/Supplementary Material, further inquiries can be directed to the corresponding authors.
